# A dataset of demographic and lifestyle risk factors for assessing chronic kidney disease development in diabetic patients

**DOI:** 10.1016/j.dib.2025.112414

**Published:** 2025-12-22

**Authors:** Ahmed Anan, Umma Tansina Arshi, Shahed Karim, Md. Kamrul Hasan, Mohammad Marufur Rahman, Taslim Taher, Rafi Nazrul Islam

**Affiliations:** a Department of Computer Science and Engineering, Ahsanullah University of Science and Technology (AUST), Dhaka 1208, Bangladesh; b Department of Nephrology, BIRDEM (Bangladesh Institute of Research and Rehabilitation in Diabetes, Endocrine and Metabolic Disorders) General Hospital, Dhaka 1000, Bangladesh

**Keywords:** Diabetes, Chronic kidney disease, Risk factor analysis, Medical care

## Abstract

Chronic Kidney Disease (CKD) is a significant comorbidity in diabetic populations, particularly in low- and middle-income countries, where early diagnosis and timely intervention remain critical healthcare challenges. This article presents a curated dataset comprising 400 diabetic patients from BIRDEM General Hospital, Dhaka, Bangladesh, with the aim of supporting early CKD detection, and healthcare planning. Among the 400 patients, 185 were diagnosed with CKD, and 215 were non-CKD cases. All individuals had been leaving with diabetes for at least ten years, enduring a consistent CKD progressing window. The dataset contains 20 variables focused on demographic and lifestyle-related risk factors, including age, gender, occupation type, BMI, family history of diabetes, hypertension, heart disease, physical activity, sleep quality, smoking, water intake, and detailed daily calorie consumption. The unique strength of this dataset lies in its longitudinal structure, which captures the health trajectories of patients over a decade. This temporal data is essential for predicting CKD progression, as it allows for the modelling of risk factors over time, a critical aspect often missed in datasets relying on single-year data. In resource-limited settings, where access to laboratory diagnostics is restricted, this dataset provides a valuable non-invasive alternative for CKD risk prediction. Given the growing global burden of CKD, especially among diabetic populations, this dataset serves as a valuable resource for researchers, healthcare providers, and policymakers seeking cost-effective, scalable strategies for early intervention and prevention.

Specifications Table

**Table:** 

Subject	Computer Sciences
Specific subject area	Chronic Kidney Disease Risk Analysis, Diabetes Data Analysis
Type of data	Table, Raw, Analyzed, Filtered, Processed.
Data collection	This dataset, collected from BIRDEM General Hospital, Dhaka, includes health records from 400 individuals, 185 diagnosed with CKD and 215 without, all of whom have been living with diabetes for over a decade. Ethical approval was obtained from the Ethical Review Committee (ERC) of the Diabetic Association of Bangladesh (BADAS), and all data used in this study were anonymized and de-identified to ensure participant privacy and confidentiality in accordance with ethical guidelines. A survey-based approach was employed using a structured questionnaire that included lifestyle-related, demographic, and non-invasive factors relevant to CKD progression. The features were selected through an iterative process that involved: features used in existing research and consultation with diabetologists and nephrologists. That ensured the chosen features were clinically relevant. Furthermore, collected data was authenticated by the nephrology department of BIRDEM.
Data source location	Institution: BIRDEM General Hospital City: Dhaka Country: Bangladesh
Data accessibility	Repository name: Mendeley doi:10.17632/hjkzgbxgv5.2 Direct URL to data: https://data.mendeley.com/datasets/hjkzgbxgv5/2 Instructions for accessing this data: The dataset is publicly available in CSV format and does not require any access controls.
Related research article	None

## 1 Value of the Data

• This dataset provides a clinically validated, non-invasive collection of demographic, lifestyle, and health-related risk factors for predicting the development of Chronic Kidney Disease (CKD) in diabetic patients. Key features include gender, job type, family history of diabetes, age, BMI, comorbidities (e.g., hypertension, heart disease), lifestyle habits (diet adherence, physical activity, smoking, water intake), and detailed dietary intake to estimate daily calorie consumption. These data points are particularly valuable in understanding how non-invasive risk factors correlate with CKD development over time.
• The dataset offers significant potential for developing and validating machine learning models for early CKD risk assessment and disease progression prediction. In initial machine learning analyses, factors such as calorie intake, BMI, age, urinary infection, duration of diabetes, and insulin intake emerged as key risk factors. For example, low calorie intake, the presence of urinary infections, and older female patients were identified as being at higher risk. Random Forest (RF) models showed promising results, achieving 86.25 % accuracy and 0.91 AUC in classifying CKD versus non-CKD individuals.
• A key feature of the dataset is its 10-year longitudinal records, which allow researchers and healthcare providers model the trajectory of CKD development and prognosticate early. This longitudinal approach facilitates exploring risk factor interactions, identifying early CKD indicators, and designing targeted intervention programs to mitigate disease progression.
• This dataset addresses critical research gaps, especially in low- and middle-income countries where healthcare resources are often limited. By focusing on non-invasive demographic and lifestyle factors, it provides an accessible and affordable alternative to traditional CKD diagnostic methods. Unlike other datasets that focus on specific regions, our dataset includes a diverse demographic from urban, semi-urban, and rural areas of Bangladesh, spanning ages 21 to 80.
• This dataset can be reused in health informatics, public health, and epidemiology to study CKD risk factors, intervention strategies, and healthcare analytics. The data could also be expanded by sourcing information from other countries and hospitals to enhance its generalizability and allow for broader trend analysis of CKD and its comorbidities across diverse populations and regions.

## 2 Background

Chronic Kidney Disease (CKD) is a major complication of diabetes mellitus, affecting approximately 30–40 % of diabetic individuals worldwide [1,2]. It contributes significantly to morbidity and mortality, particularly in low-and middle-income countries, where access to renal replacement therapies is limited. In countries such as Bangladesh, fewer than 10 % of patients requiring dialysis or kidney transplants receive them, highlighting the critical need for early detection strategies [3].

Given the complexity and wide-ranging impact of CKD, robust datasets are essential for accurate research and risk assessment. However, existing datasets used for CKD prediction, such as the Chronic Kidney Disease dataset [4] and datasets in these notable works [5,6] often suffer from limitations, including lack of trend analysis, insufficient demographic representation, and limited lifestyle parameters. These shortcomings hinder the generalizability and applicability of these datasets to broader populations and modern healthcare contexts.

This dataset addresses these gaps by incorporating a wider range of demographic and lifestyle variables, along with 10 years of longitudinal data for each patient. This expanded feature set enhances the dataset’s relevance and utility for CKD research in diabetic populations, providing accessible risk factors ideal for developing robust predictive models and supporting prevention and management efforts.

## 3 Data Description

Patients included in the study had been living with diabetes for over a decade and had health records available for analysis. Table 1 presents all the used features and their descriptions. CKD positive cases were identified through confirmed diagnoses based on medical records. The interval between diabetes diagnosis and CKD onset was also recorded. Patients with incomplete medical records were excluded from the data collection process.

**Table 1:** Description of feature attributes.

Sl. No.	Risk Factors	Data Type	Description
1	Gender	Categorical	Male (M), Female (F)
2	Job	Categorical	Level of physical exertion during working life: **Normal** (Involves minimal physical effort as part of daily tasks, e.g., Banker); **Intermediate** (Includes moderate physical activity as part of the job, e.g., delivery worker; **Heavy** (Requires high exertion throughout the day, e.g., construction worker)
3	Family history of diabetes	Boolean	Whether the patient’s parents had diabetes: Yes (1), No (0)
4	Duration of diabetes	Integer	Number of years the patient has been diagnosed with diabetes
5	Age (years)	Integer	Age of the patient in years
6	BMI (kg/m2)	Float	kg/m²
7	Follow suggested Diet	Boolean	Whether the patient consistently managed their diabetes by maintaining proper lifestyle: Yes (1), No (0)
8	Take Medicine for Diabetes	Boolean	Whether the patient takes diabetes medication: Yes (1), No (0)
9	Take Insulin	Boolean	Whether the patient takes insulin: Yes (1), No (0)
10	Hypertension	Boolean	Whether the patient has hypertension (blood pressure consistently at or above 140/90 mm ¦Hg.): Yes (1), No (0)
11	Heart Disease	Boolean	Whether the patient has heart disease: Yes (1), No (0)
12	Sleep	Boolean	Typical sleep duration: (Inadequate(*<*7hours), Adequate(7–-9hours)): Sufficient (1), Insufficient (0)
13	Water Consumption	Boolean	Level of water intake (Inadequate (less or equal to 2 litres/day) adequate (>2 litres/day)): Sufficient (1), Insufficient (0)
14	Smoke	Boolean	Whether the patient smokes: Yes (1), No (0)
15	Zarda, Betel Leaf	Boolean	Whether the patient consumes Zarda or betel leaf: Yes (1), No (0)
16	Walk Regularly	Boolean	Whether the patient walks regularly (at least 30 min a day): Yes (1), No (0)
17	Urination Properly	Boolean	Whether the patient can urinate properly without any discomfort: Yes (1), No (0)
18	Urinary Infection	Boolean	Whether the patient has urinary infections Yes (1), No (0)
19	Pain killer	Boolean	Whether the patient has to take pain killers in regular basis: Yes (1), No (0)
20	Calorie Intake (Cal)	Integer	Average daily calorie intake
**Output**	CKD	Boolean	Yes (1), No (0)

All data for CKD patients were collected retrospectively, focusing on the diabetic period prior to the onset of CKD. For diabetic non-CKD patients, data were collected from the period between current and past 10 years, reflecting their current conditions and behaviors.

**Fig. 1:**
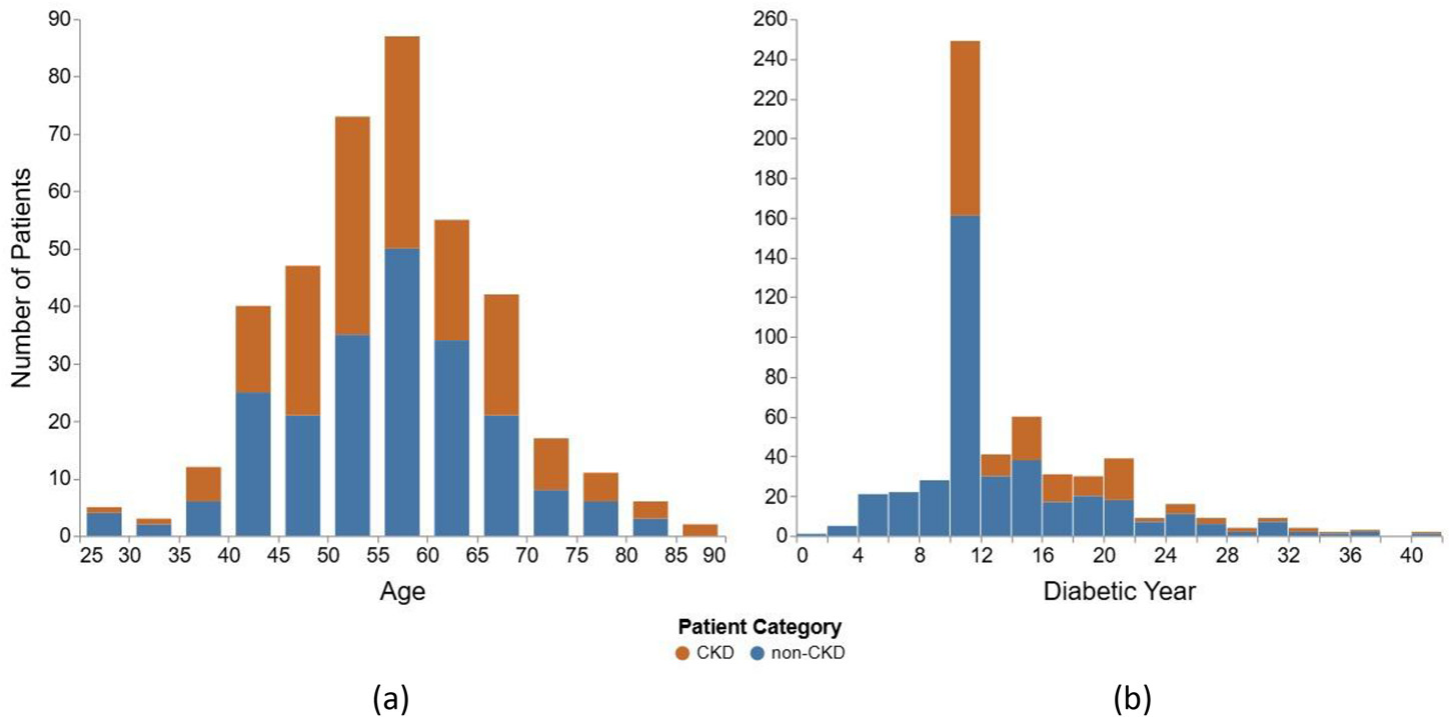
Age-wise Distribution of CKD and Non-CKD Patients (a) and Duration of Diabetes Among CKD and Non-CKD Patients (b).

The dataset includes 400 diabetic patients, of whom 185 (46.25 %) were diagnosed with CKD while 215 (53.75 %) did not have CKD. Among the CKD individuals, 113 were female and 72 were male. The mean age of CKD patients (54.17 years) was significantly higher than those without CKD (49.10 years). A breakdown by age reveals that 87.6 % of CKD cases occurred in individuals over 45 years. In contrast, only 8 CKD patients were younger than 40. Fig. 1(a) depicts the distribution of CKD and non-CKD patients with respect to age. Additionally, it is noteworthy that most CKD patients had been living with diabetes for over 10 years during the survey which can be observed in Fig. 1(b).

While CKD manifestation remains lower in younger diabetic individuals, early onset is not uncommon, with some patients in their late 30 s already showing signs of kidney dysfunction.

Among the 185 CKD patients, 140 (75.68 %) reported sedentary or non-active lifestyles. When stratified by job type 31 (16.76 %) CKD patients did moderate physical activity as part of daily tasks, and only 14 (7.57 %) as heavy labor. In contrast, more active employment and routine physical activity were associated with a lower prevalence of CKD. Although BMI values were relatively similar 24.81 kg/m² for CKD patients versus 24.03 kg/m² for non-CKD individuals, other clinical and lifestyle risk factors revealed more marked differences. Hypertension, a well-established contributor to kidney dysfunction, was present in 30.81 % of CKD patients. Sleep quality and hydration habits also varied: 45.41 % of CKD individuals reported insufficient sleep, compared to 35.35 % in the non-CKD group; adequate water intake was noted in 88.11 % of CKD patients versus 92.56 % of non-CKD patients. Smoking was more common among CKD patients (16.22 %) than among those without CKD (10.70 %), whereas the use of Zarda or betel leaf was nearly equal across both groups (23.24 % ¦vs. 24.19 %). Furthermore, a family history of diabetes was reported by 43.78 % of CKD patients, indicating a potential hereditary component in disease susceptibility.

Mentioned regional groups in Table 2 differ in lifestyle, occupational exposure, and healthcare accessibility, which may influence CKD risk. Urban participants, who constitute the majority of the sample, are more likely to experience sedentary lifestyles and higher intake of processed foods, increasing metabolic risk factors such as obesity and hypertension. Rural participants often engage in physically demanding work but may have limited access to healthcare and greater exposure to environmental contaminants, potentially elevating CKD risk through non-metabolic pathways. Semi-urban participants represent a transitional group with mixed exposure to both urban and rural risk patterns.

**Table 2:** Regional distribution and CKD prevalence.

Region	Patients (n)	Percentage	CKD Patients	CKD %
Urban	215	53.75 %	101	46.98 %
Rural	100	25.00 %	44	44.00 %
Semi-urban	85	21.25 %	40	47.06 %

**Fig. 2:**
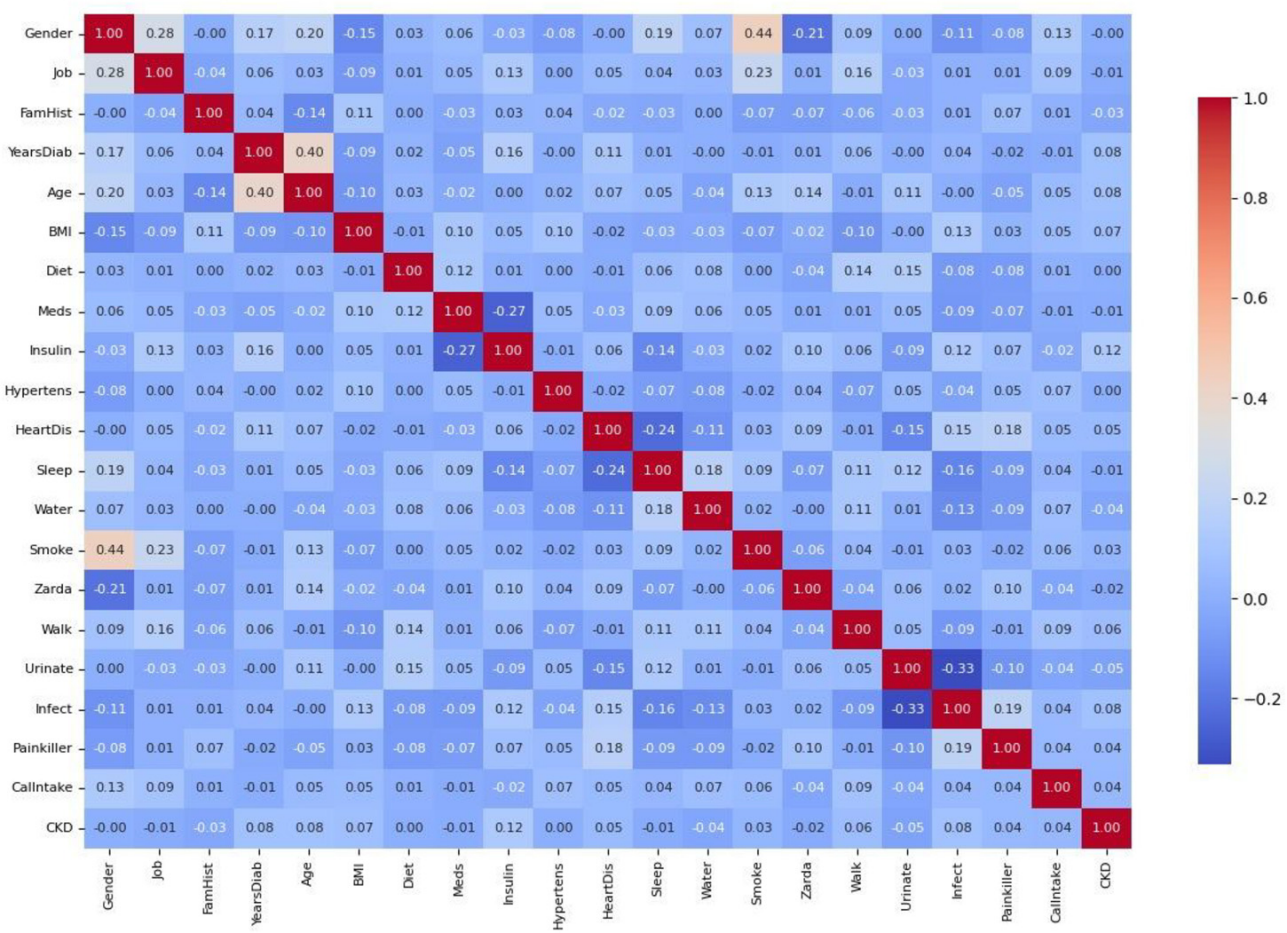
Correlation matrix of clinical and medical parameters.

A correlation heatmap illustrated in Fig. 2 was generated to assess interrelationships among the 20 risk factors. A strong positive correlation (0.40) was observed between age and diabetic years, and moderate correlations were found between gender and smoking (0.44), and gender and job type (0.28). In contrast, negative correlations were recorded between urination regularity and urinary infection (−0.33), and between insulin and medicine intake (−0.27).

## 4 Experimental Design, Materials and Methods

This section outlines the comprehensive strategy and methodology adopted for data collection and validation. All individuals included in the study had been living with diabetes for over a decade and possessed complete health records. Approximately 450 patients participated in the survey between March and June 2024, from which 400 records were finalized after applying quality control measures. The participant pool reflected a diverse cross-section of the Bangladeshi diabetic population, including individuals from urban, semi-urban, and underprivileged backgrounds across different geographic regions. This diversity enriches the dataset’s demographic scope and enhances its utility for broader CKD research and predictive modelling.

### 4.1 Limitations of existing data sets

Existing datasets used for CKD prediction, such as the chronic kidney disease dataset, often lack diverse demographic and lifestyle-related features, instead focusing on conventional, clinically dependent risk factors that require costly diagnostics, limiting their use in resource-constrained settings. Other notable datasets referenced in works [5,6] face similar issues, including limited demographic representation, absence of comprehensive lifestyle data, and lack of trend analysis over time.

Predicting CKD based on a single point of data is highly challenging, as the disease develops gradually and depends on long-term risk factor trajectories [7]. The absence of longitudinal information in existing datasets restricts their ability to capture the full picture of CKD progression, limiting their relevance in real-world clinical and research applications.

### 4.2 Data collection methodology

**Fig. 3:**
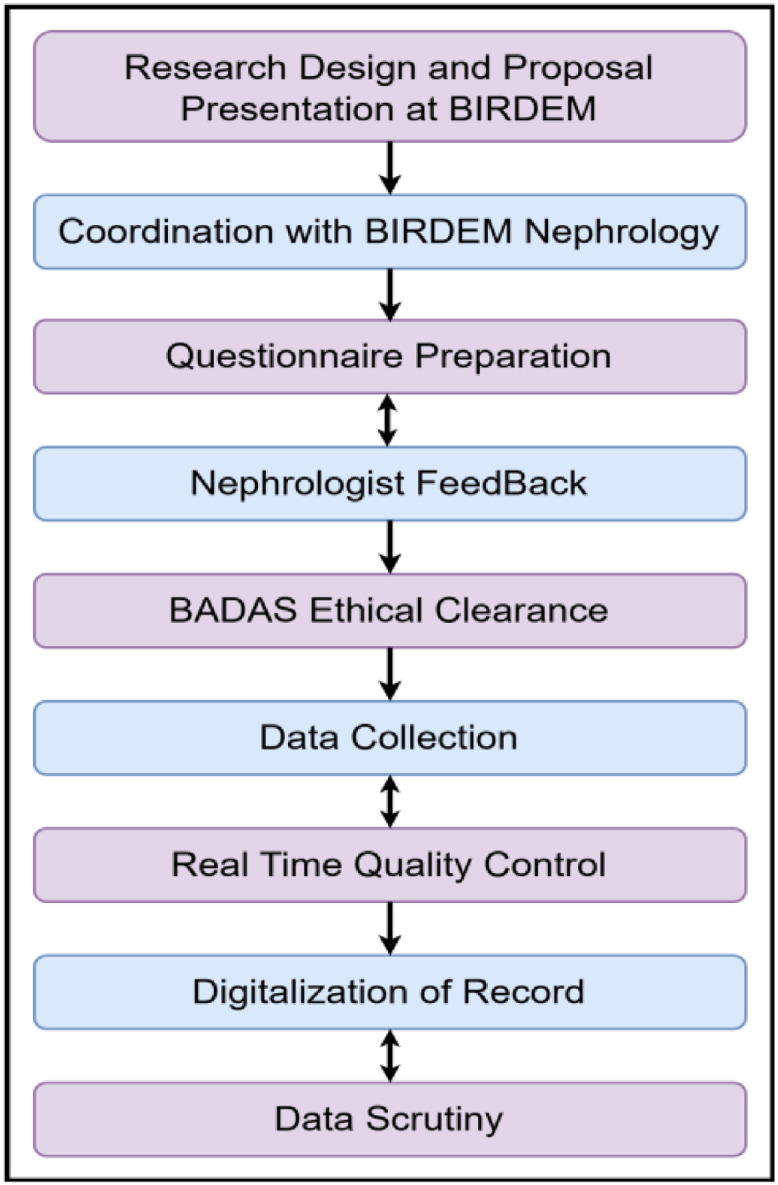
Structured workflow of the data collection and validation process.

The data collection phase of this study was conducted in collaboration with BIRDEM General Hospital, Dhaka [8], and was approved by the Ethical Review Committee (ERC) of the Diabetic Association of Bangladesh (BADAS). Following ethical approval, a formal collaboration was established with the Nephrology Department of BIRDEM, in accordance with ERC guidelines requiring the direct involvement of a qualified nephrologist in the data collection process. The structured workflow is shown in Fig. 3.

We collaborated with a senior nephrologist from BIRDEM, who supervised the entire process. Together, we developed a structured questionnaire designed to capture non-invasive, lifestyle-related, and demographic risk factors relevant to CKD progression. To strengthen the clinical validity of the questionnaire, we followed a structured multistage validation procedure under the direct supervision of our appointed nephrologist. The initial version of the questionnaire was developed based on an extensive review of high-impact literature on diabetic CKD and was subsequently reviewed by the nephrologist to ensure clinical relevance. Two pilot tests were then conducted with small groups of diabetic patients during routine clinic sessions. During these pilot rounds, we assessed question clarity, patient comprehension, response consistency, and clinical interpretability. Based on observed challenges and patient feedback, several items particularly related to dietary habits, occupational activity, and lifestyle factors were refined or reformulated. After two iterations of testing and revision, the questionnaire was finalized and approved by the supervising nephrologist for full-scale data collection. The complete structured questionnaire is provided in the Supplementary Materials.

Data were collected through in-person interviews conducted during routine hospital visits. Eligibility criteria required that participants had lived with diabetes for at least ten consecutive years and possessed a complete diabetes record book documenting their annual clinical history. During interviews, questionnaire responses were cross-checked with the patients’ record books. All data collected were anonymized at the point of entry, and no personally identifiable information was recorded or retained and unique identifiers were not assigned.

Following collection, the data underwent a multi-stage validation process. The finalized dataset was reviewed and authenticated by the Nephrology Department (BIRDEM), ensuring clinical accuracy and consistency with ethical standards.

### 4.3 Data preprocessing

A strict completeness criterion was applied during screening: only patients with fully documented 10-year diabetes record books and complete questionnaire responses were eligible for inclusion. Approximately 50 individuals were excluded due to missing or irregular documentation, incomplete longitudinal entries, or unreliable responses to key lifestyle or dietary questions. These exclusions were determined prior to digitization based on predefined criteria required to maintain the continuity and integrity of the longitudinal structure.

After applying these criteria, 400 patient records remained, each containing complete data across all variables and all 10 years. Because the final dataset contained no missing values, no statistical imputation was required. The selected records were systematically cleaned, encoded, and formatted to ensure consistency. Age and weight were updated annually, and Body Mass Index (BMI) was computed using the mean values across the observation period.

No confounding adjustment or variable selection was performed, as the purpose of this work is to publish the dataset in its original and complete form. General associations among predictors were visually inspected using the correlation matrix (Fig. 2), which showed expected conceptual overlaps (e.g., age with BMI, dietary intake with BMI). All variables were retained to ensure full transparency and allow future researchers to apply their own confounding-control methods as appropriate to their analyses.

#### 4.3.1 Dietary calorie standardization

Daily Calorie Intake was computed using the official dietary portion values provided by the Bangladesh Diabetic Association (BADAS) Dietary Guidelines [9], which specify standardized household-level caloric values (e.g., 1 cup cooked rice = 150 kcal, 1 bread/roti = 75 kcal, 1 serving fish/meat = 120 kcal, 1 egg = 130 kcal).

Patients reported their typical daily diet using informal quantities (e.g., “two plates of rice,” “three parathas”). All such responses were first converted into BADAS-standard portion equivalents (e.g., 1 plate of rice ≈ 2 cups), after which the corresponding caloric values were assigned. Items consumed weekly (e.g., sweets, soft drinks) were converted to daily averages by dividing total weekly calories by seven.

The total daily caloric intake for each participant was then computed by summing the standardized caloric contributions from all food categories. This procedure ensured consistent, guideline-based estimation of dietary energy intake across all patients.

## Limitations

One of the primary limitations of this study is the relatively small sample size, which was influenced by time constraints and limited human resources during the data collection phase. Additionally, participant reluctance often due to a lack of awareness regarding the purpose of research and the time required for survey participation further restricted the number of respondents. Another significant challenge was that many eligible patients did not have their health record books during the interview sessions, leading to their exclusion from the dataset due to the absence of longitudinal documentation. These factors collectively limited the scope of data inclusion, which may affect the broader generalizability of the findings. However, the curated dataset remains a valuable contribution to this research domain. Given that CKD often develops within 7–8 years of diabetes onset, the dataset’s longitudinal structure can serve as a strong foundation for developing more effective and accessible risk prediction models.

## Ethics Statement

The data collection and validation process strictly adhered to established ethical guidelines, with all research protocols approved by the Ethical Review Committee (ERC) of the Diabetic Association of Bangladesh (BADAS) under reference number **BADAS-ERC/EC/24/11**. The study maintained rigorous standards of confidentiality and data protection, ensuring that no personally identifiable information was disclosed or accessed without authorization. Informed written consent was obtained from all participants prior to their involvement. Each participant was thoroughly briefed about the objectives of the study, the voluntary nature of their participation, the confidentiality of their responses, and their right to withdraw at any point without any consequences. Participation was entirely voluntary and based on a clear understanding of the research scope. All ethical principles including respect for autonomy, privacy, and responsible data sharing were carefully observed throughout the study to uphold the integrity and rights of the participants.

## CRediT Author Statement

**Ahmed Anan:** Conceptualization, Methodology, Visualization, Investigation, Data Curation, Formal Analysis, writing original draft, and editing. **Umma Tansina Arshi:** Conceptualization, Methodology, Investigation, Data Curation, Formal Analysis, writing original draft, and editing. **Shahed Karim:** Data Curation. **Md. Kamrul Hasan:** Data Curation. **Mohammad Marufur Rahman:** Supervision, Conceptualization, Methodology, Investigation, Data Validation, review, and editing. **Taslim Taher:** Supervision, review. **Rafi Nazrul Islam:** Supervision, Investigation, Data Validation, review.

## Data Availability

Mendeley DataA Dataset on Demographic and Lifestyle Factors for Prognosticating Chronic Kidney Disease Progression in Diabetic Patients (Original data).

## Appendix Supplementary Materials

Image, application 1

Image, application 2

Image, application 3
