# Supplementary figures and images for: A dataset of demographic and lifestyle risk factors for assessing chronic kidney disease development in diabetic patients
Source: Data Brief. 2025 Dec 22;64:112414. doi: 10.1016/j.dib.2025.112414 (PMC12834833; doi:10.1016/j.dib.2025.112414)

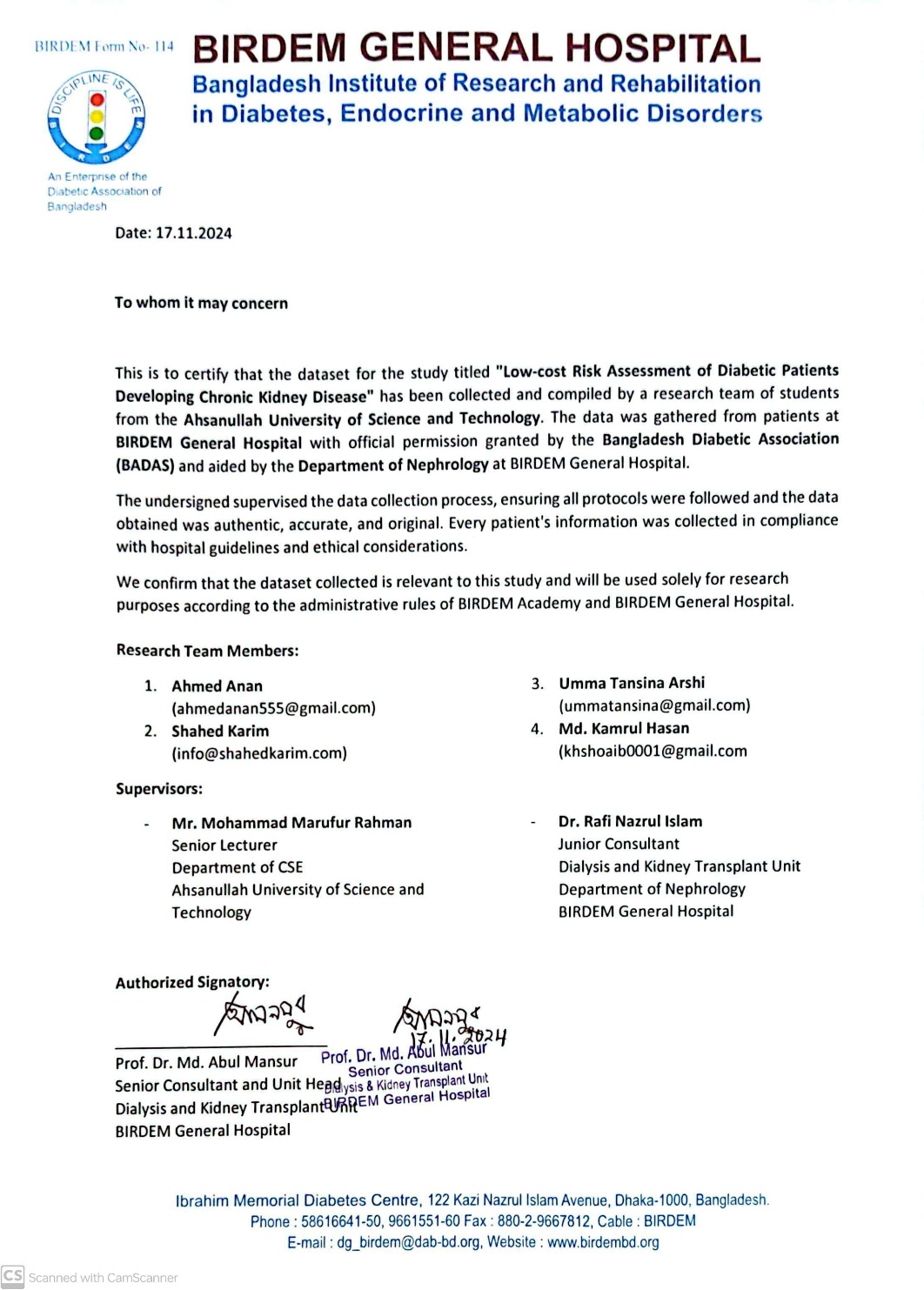


Figure 1: Data authentication certificate.


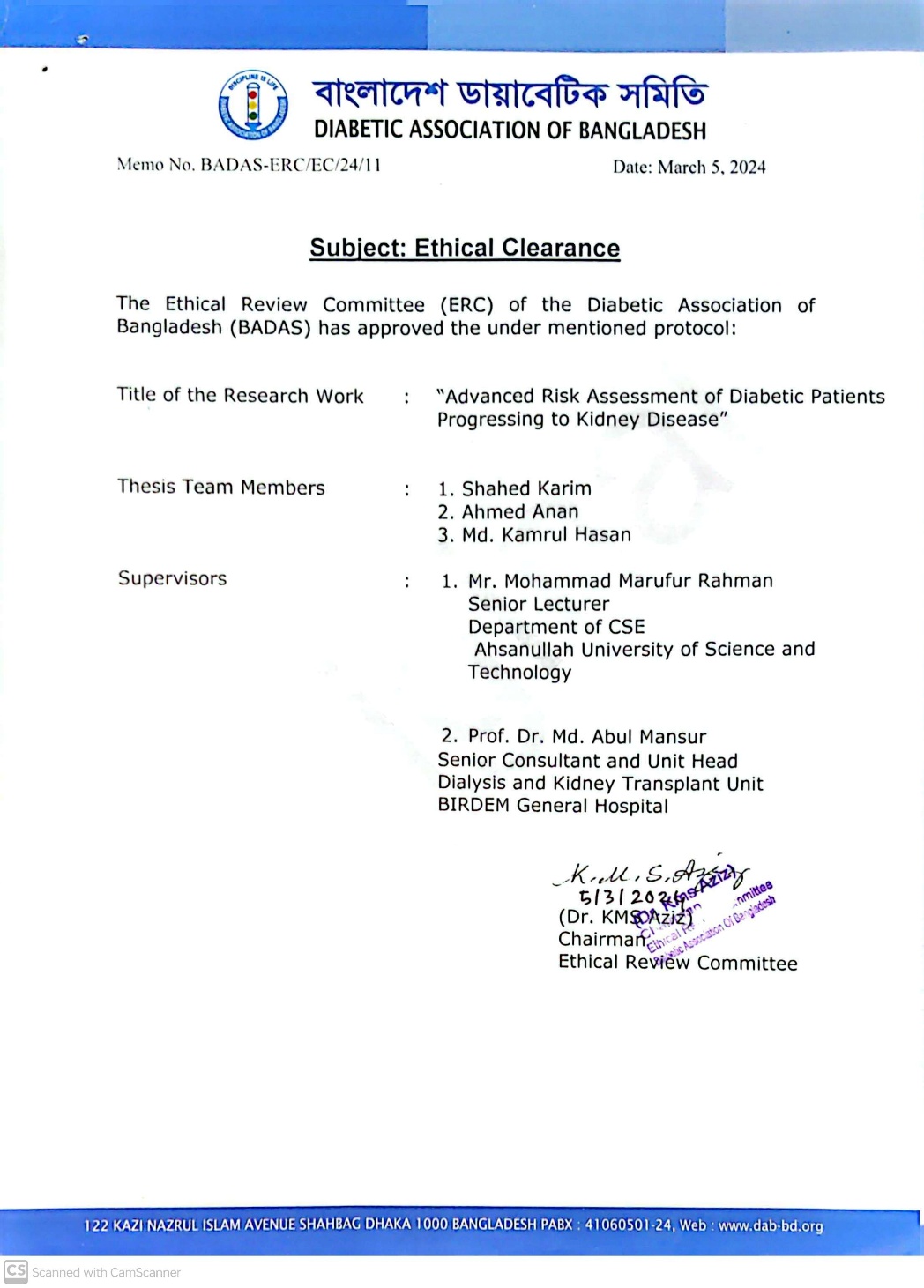


Figure 2: Ethical Clearence Certificate.

Supplement: Supplementary file 1 [file mmc1.docx]
