# Supplementary material for: A dataset of demographic and lifestyle risk factors for assessing chronic kidney disease development in diabetic patients
Source: Data Brief. 2025 Dec 22;64:112414. doi: 10.1016/j.dib.2025.112414 (PMC12834833; doi:10.1016/j.dib.2025.112414)
Supplement: Supplementary file 2 [file mmc2.docx]

**RESEARCH TEAM AND STUDY DETAILS**

**Principal Investigators:**

- Ahmed Anan (BSc)
- Umma Tansina Arshi (BSc)
- Shahed Karim (BSc)
- Kamrul Hasan (BSc)
- Marufur Rahman (Senior Lecturer)

**Institutional Affiliation:**

This research study is being conducted by the Department of Computer Science and Engineering of Ahsanullah University of Science and Technology. The study has been approved and authorized by the respective department and institutional review board.

**Study Objectives:**

The primary objective of this research is to develop an artificial intelligence-based predictive model for diabetic patients who are at risk of developing kidney disease. By analyzing demographic and lifestyle factors through advanced machine learning techniques, this study aims to:

1. Identify risk factors associated with diabetic kidney disease
2. Develop predictive algorithms for early detection
3. Create a risk assessment tool for healthcare providers
4. Contribute to personalized medicine approaches for diabetic patients

**Study Procedures:**

The data collection process will involve a single interview session lasting approximately 10-12 minutes.

**Data Collection Protocol:**

**Primary Assessment:**

- Participants who consent to participate will be asked questions through a complete and structured questionnaire consisting of 15-20 questions.

**Information Collected:**

- **Demographic Information:** Age, gender, occupation
- **Medical History:** Duration of diabetes, family history of diabetes and kidney disease
- **Current Health Status:** Blood pressure, current medications
- **Lifestyle Factors:**
  - Dietary habits and nutritional patterns
  - Physical activity and exercise routine
  - Water intake and hydration habits
  - Sleep patterns and quality
  - Smoking and alcohol consumption history
  - Stress levels and management
- **Comorbidities:** Presence of other medical conditions

**Inclusion Criteria:**

- Diagnosed diabetic patients with confirmed kidney disease
- Ability to provide informed consent and have record books with them
- Willingness to participate in the interview process

**Follow-up and Monitoring:**

- Following data collection, no additional follow-up visits will be required from participants. This is a one-time data collection study.

**Adverse Event Reporting:**

- In the unlikely event of any adverse occurrences during the study, the research team will assume full responsibility and provide appropriate medical care and intervention as necessary.

**Confidentiality and Data Protection:**

- All collected data will be used exclusively for research purposes and will be stored with strict confidentiality measures. All data will be de-identified and stored securely in accordance with institutional data protection policies.

**Right to Withdrawal:**

- You have the absolute right to withdraw from this study at any time without providing any reason. Your withdrawal will not affect your current medical treatment or access to healthcare services in any way. You will not be subjected to any penalty or loss of benefits for withdrawing from the study.

**Compensation and Incentives:**

- No financial compensation or incentives will be provided for participation in this research study. Any costs incurred during routine medical care will be handled according to standard hospital policies and will not be influenced by study participation.

**Risks and Benefits:**

- This study involves no risk, as it only requires providing information about your lifestyle and medical history. There are no experimental procedures or interventions involved. The potential benefits include contributing to medical knowledge that may improve future care for diabetic patients with kidney disease.

We sincerely appreciate your cooperation and participation in this research study.

**PARTICIPANT ACKNOWLEDGMENT:**

I acknowledge that I have read and understood the information provided about this research study. I have had the opportunity to ask questions, and all my questions have been answered to my satisfaction. I understand that my participation is voluntary and that I may withdraw at any time without penalty.

**Participant Name:** _________________________________

**Participant Signature:** _________________ **Date:** _________

**Researcher Name:** _________________________________

**Researcher Signature:** _________________ **Date:** _________

**Witness Name:** _________________________________

**Witness Signature:** _________________ **Date:** _________
