# Supplementary material for: A dataset of demographic and lifestyle risk factors for assessing chronic kidney disease development in diabetic patients
Source: Data Brief. 2025 Dec 22;64:112414. doi: 10.1016/j.dib.2025.112414 (PMC12834833; doi:10.1016/j.dib.2025.112414)
Supplement: Supplementary file 3 [file mmc3.docx]

**Title: *A Dataset of Demographic and Lifestyle Risk Factors for Assessing Chronic Kidney Disease Development in Diabetic Patients.***

**Data collection questionnaire**

**CKD:**

**Yes**

**No**

|  |  |  |  |
| --- | --- | --- | --- |

**Serial No:**

- **The patient’s place of residence**

a. City b. Urban c. Village

- **Did the patient use to consume gastric medicine (anti-ulcer)?**

a. Yes, ..........................mg b. No

**1. Age:**

…………………… Years

**2. Duration of diabetes:**

…………………… Years

starting year ……………………

**3. Family history of diabetes:**

a. Have/ had (relationship with patient) ……………………

b. None

**4. Duration of kidney disease:**

…………………… Years

starting year ……………………

**5. Family history of kidney diseases:**

a. Have/ had (relationship with patient) ……………………

b. None

**6. Gender:**

a. Male b. Women c. Others

**7. Height:**

…………………… feet /inch /centimeter

**Weight:**

CKD

Diabetes

…………………… Kilogram

**8. Level of physical exertion during working life:**

a. General, profession ……………………

b. Medium, profession ……………………

c. Heavy, profession ……………………

**9. How they manage diabetes:**

| Year |  |  |  |  |  |  |
| --- | --- | --- | --- | --- | --- | --- |
| Controlling Diet |  |  |  |  |  |  |
| Taking Medicine |  |  |  |  |  |  |
| Taking Insulin |  |  |  |  |  |  |

**10. Blood pressure:**

CKD

Diabetes

a. Controlled: (100-140 mmHg and/or 60-90 mmHg)
 b. Uncontrolled:

Grade-1:(141-159 mmHg and/or 90-99 mmHg)

Grade 2:(160-179 mmHg and/or 100-109 mmHg)

Grade-3:( ≥180 mmHg and/or ≥110 mmHg)

1. Low: (<100mmHg - <60mmHg)

**11. Heart disease:**

Diabetes

CKD

a. Yes

b. No

**12. Sleep duration:**

CKD

Diabetes

a. Inadequate (<7 hours)

b. Adequate (7-9 hours)

c. Excessive (>9 hours)

**13. Water consumption levels:**

Diabetes

CKD

a. Inadequate (≤2 liters /day)

b. Adequate (≥2 liters/day)

**14. Smoker?**

-> Cigarettes, pipes (**five sticks or above per day for four to five years):

a. Yes, for how many years …………………… amount, how many times a day……………………

b. No

-> Smokeless tobacco (Zarda, BetelLeaf):

a. Yes, for how many years …………………… amount, how many times a day……………………

b. No

**15. Regular walking (at least 30 minutes a day, five days a week):**

a. happens /used to happen

Diabetes

CKD

b. doesn't happen /used not to happen

c. Irregular

**16.**  **Timely urination:**

CKD

Diabetes

a. happens/ used to happen

b. doesn't happen / used not to happen

**17. Urinary infection:**

CKD

Diabetes

a. Yes, duration ……………………

b. No

**18. Excessive painkiller consumption (2/3times a month):**

a. happens /used to happen

CKD

Diabetes

b. doesn't happen / sed not to happen

**19. Food consumption/day:**

| Types of food | Name of food | Amount/Day | Amount/Week | Calorie/Day |
| --- | --- | --- | --- | --- |
| Carbohydrates | Rice, Bread, Parath, Khichuri, Puffed Rice |  |  |  |
|  | Others |  |  |  |
| Protein | Egg |  |  |  |
|  | Fis, Chicken, Beef, Mutton |  |  |  |
|  | Others |  |  |  |
| Milk and Dairy products | Milk |  |  |  |
|  | Yogurt, Sweet, curd |  |  |  |
| Drinks | Cold Drinks |  |  |  |
